# Isolation of an *Escherichia coli *K4 *kfoC *mutant over-producing capsular chondroitin

**DOI:** 10.1186/1475-2859-9-34

**Published:** 2010-05-17

**Authors:** Anna Zanfardino, Odile F Restaino, Eugenio Notomista, Donatella Cimini, Chiara Schiraldi, Mario De Rosa, Maurilio De Felice, Mario Varcamonti

**Affiliations:** 1 Department of Structural and Functional Biology, University of Naples Federico II, Naples, Italy; 2 Department of Experimental Medicine, Second University of Naples, Italy

## Abstract

**Background:**

Chondroitin sulphate is a complex polysaccharide having important structural and protective functions in animal tissues. Extracted from animals, this compound is used as a human anti-inflammatory drug. Among bacteria, *Escherichia coli *K4 produces a capsule containing a non-sulphate chondroitin and its development may provide an efficient and cheap fermentative production of the polysaccharide.

**Results:**

A random *N*-methyl-*N*'-nitro-*N*-nitrosoguanidine mutagenesis was performed on *E. coli *K4 to isolate mutants showing an increased production of chondroitin. Several mutants were isolated, one of which, here named VZ15, produced about 80% more chondroitin than the wild type *E. coli*. We found that the mutant has a missense mutation in the codon 313 of *kfoC*, the gene encoding chondroitin polymerase (K4CP), with a change from arginine to glutamine. A docking analysis to explain the increased productivity of the K4CP enzyme is presented.

**Conclusion:**

The enhanced chondroitin production by the *E. coli *K4 mutant reported here shows the validity of the strain improvement strategy for more cost-friendly fermentative processes in the production of this pharmaceutically important but so-far expensive polysaccharide.

## Background

Chondroitin sulphate (CS) is a glycosaminoglycan formed by a repeated disaccharide unit consisting of D-glucuronic acid (GlcUA) and *N*-acetyl-D-galactosamine (GalNAc). The disaccharides are often sulphated in position 4 or 6 of the GalNAc, but other sulphation patterns have also been observed. Human proteoglycans having CS chains are abundant in cartilage [1], aorta, skeletal muscle, eye [2], lung [3], and brain [4,5]. They are synthesized intracellularly and secreted to form a macromolecular complex directed to the extracellular matrix or localized at the cell surface. Biosynthesis of the CS chain of CS-proteoglycans occurs in the Golgi apparatus [6]. During polymerization, a variety of sulfotransferases [7] catalyze the sulphation at various positions.

CS can be used as a nutraceutical substance, i.e. nutritional supplement with proven pharmaceutical properties and efficacy. In fact a number of studies have been focused on the biochemical activities of orally administered exogenous CS [8]. It has been suggested [9] that its anti-inflammatory and chondro-protective properties result from an increase in the biosynthesis of connective tissue components, such as hyaluronic acid, and an increase of the viscosity of the synovial fluid at disease sites. Based on meta-analysis of various clinical studies, CS is presently recommended in Europe as a symptomatic slow-acting drug in the treatment of knee osteoarthritis (OA). In vitro and in vivo studies have shown that CS regulates the formation of new cartilage by stimulating the chondrocyte synthesis of collagen, proteoglycans, and hyaluronan [10].

Until now extraction of commercial chondroitin sulphate from animal tissues has required complex purification procedures owing to risks of viral and prionic contaminations (e.g. extraction from pigs) and high costs, besides scarcity of row materials due to protection of animal species under extinction risk (e.g. sharks and chondrichthyes). Recently, a growing interest towards the discovery of novel sources of chondroitin and production processes has arisen. Some other animal species produce CS chains, including squid [11], king crab [12] and *Caenorhabditis elegans *[13].

Also bacteria can produce chondroitin chains, although not sulphated. Indeed, Gram-negative bacteria such as *Escherichia coli *K4 contain polysaccharides similar to CS chains in their capsule. Using this feature to its advantage, the K4 strain of *Escherichia coli *promotes its invasiveness and pathogenicity to host cells by masking its presence and avoiding antibody response. The K4 capsule contains a polysaccharide made of chondroitin [GlcUA β(1-3)-GalNAc β(1-4)]_n _to which fructose is β-linked at position C-3 of the GlcUA residue [14]. During synthesis the addition of fructose branches occurs after the chondroitin elongation [15]. The enzyme that catalyzes elongation of the chondroitin chain in *E. coli *K4 was identified as protein K4CP, the product of the *kfoc *gene [16]. However, the amount of chondroitin produced by K4 is not enough to allow its use as a potential industrial platform for the production of a chondroitin-like polymer. A very recent work has demonstrated that the quality of carbon source and growth conditions can greatly affect polysaccharide production [17].

In this work we performed an *N*-methyl-*N*'-nitro-*N*-nitrosoguanidine random mutagenesis to select *E. coli *K4 mutants that produce an increased amount of the polysaccharide and focused our attention on a specific one.

## Results and discussion

### Random mutagenesis by N-methyl-N'-nitro-N-nitrosoguanidine

The *Escherichia coli *K4 chromosome was random-mutagenized in vivo in order to induce mutations to be selected for higher production of capsule polysaccharide (CPS) and, consequently, of chondroitin. In fact, our hypothesis was that a larger capsule should also contain higher amounts of K4-chondroitin.

The mutagen used was *N*-methyl-*N*'-nitro-*N*-nitrosoguanidine (MNNG), whose action is to induce transitions in DNA molecules. Its working concentration was 50 μg/ml, allowing 75% survival of bacterial cells.

After MNNG treatment, cells were plated on agar medium containing ampicillin and chloramphenicol (2,5 and 3.0 μg/ml respectively, the MIC for wild type K4 when both antibiotics were present). The choice of two antibiotics was made in order to exclude mutants resistant to a single one, since single resistance could be antibiotic-related and not dependent on capsule dimension. Plates were incubated at 30°C in order to prevent lethality of temperature-sensitive mutations. Colonies able to grow on double antibiotic selective medium were considered as putative candidates to produce a capsule larger than that of the wild type, which would prevent antibiotics activities [18].

### Colorimetric assay with indole-3-acetic-acid

To check the ability of mutants to produce enhanced amounts of K4-CPS, 100 double-antibiotic resistant colonies, the control strain K4 (wild type) and *DH5α *(non-capsulated strain) were tested by a colorimetric assay. This assay was based on the estimation of colour developed on plates by colonies following a reaction between the C3-fructose residue of glucuronic acid and 0.05% indole-3-acetic acid.

**Figure 1:**
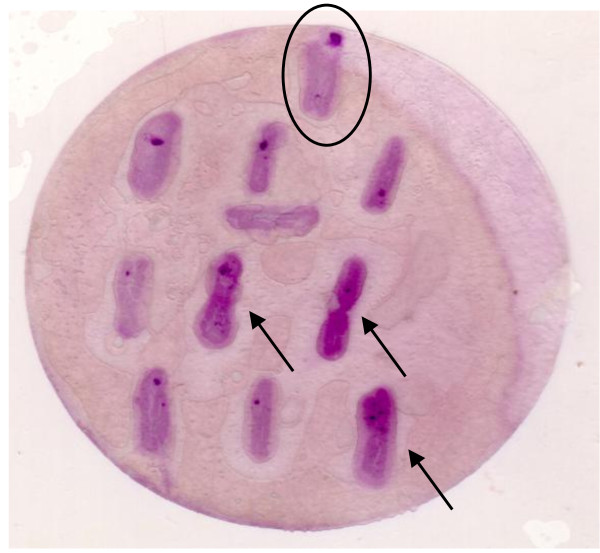
**Colorimetric assay**. Colorimetric assay of capsule polysaccharide production performed on wild type *E. coli *K4 (circled) and some of the selected mutants developing an intense colour (arrows).

From these experiments we observed that: i) colonies of the negative control strain *DH5α *remained unstained (data not shown); ii) those of the wild type strain K4 showed a moderately intense violet colour (Fig. 1); iii) some mutant colonies developed a violet colour distinctively more intense than that of the wild type bacteria (Fig. 1). Cells from these mutant colonies were considered as good candidates producers of enhanced amounts of CPS, and thus chosen for further analyses.

### Chondroitin production

Mutants selected from the previous assays were analyzed for their quantitative CPS production. Cells of the mutants and of the K4 wild type strain were grown in K4-medium and after centrifugation their supernatants were concentrated using centricon filters with 10 kDa cut-off and analyzed by the carbazole assay followed by capillary electrophoresis (see Methods).

The carbazole assay showed that 45 mutants had an enhanced CPS production after 24 hours with respect to K4 wt strain; mutants showing a minimum of 20% higher production compared to the wild type were taken under consideration for further studies. These 15 mutants were subjected to a screening by capillary electrophoresis in order to quantify the specific chondroitin moiety produced by each [19].

**Figure 2:**
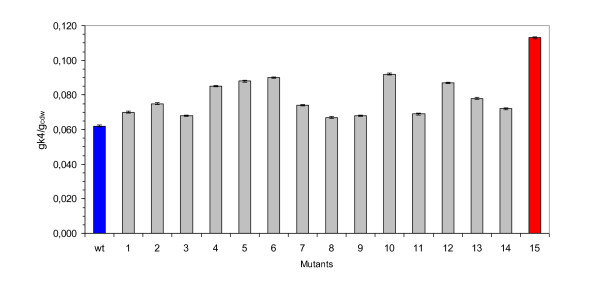
**Polysaccharide production**. Yield for K4-polysaccharide on biomass formed by 15 mutants and the wild type K4 strain. Wild type K4 and VZ15 mutant are evidenced in blue and red, respectively.

The wt strain released 0.117 ± g·L^-1 ^of polysaccharide in the supernatant at the 24^th ^hour of growth while several mutants showed a slightly higher production. One mutant, VZ15, released in the medium 0.214 ± 0.005 g·L^-1 ^of K4 polysaccharide after 24 hours, and showed a yield for polymer on biomass formed significantly higher than that of the wt strain (0.113 gK4·g_cdw_^-1 ^vs. 0.062 gK4·g_cdw_^-1^) (Fig. 2). The K4-polysaccharide yield value of VZ15 was 82% higher than that reported for the wild type strain. Thus, the VZ15 mutant was selected as a good over-producer strain.

It should be noted that the growth curve of VZ15 was coincident to that of the wild type (data not shown).

### kfoC characterization

**Figure 3:**
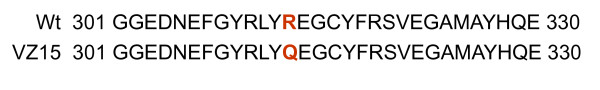
**Amino acidic sequence**. 301-330 amino acidic sequence of K4CP protein from wild type and VZ15 mutant.

Among the possible candidate genes responsible for the phenotype of CPS over-production in VZ15, we decided first of all to consider the *kfoC *gene, which belongs to the region 2 of the Kps cluster and encodes a protein known as chondroitin polymerase (K4CP), whose structure has recently been solved by X-ray crystallography [20]. In order to understand if the CPS increase in VZ15 was associated to a mutation in the *kfoC *gene, we cloned the *kfoC *gene of VZ15 in a plasmid vector and sequenced it. After sequencing we mapped a G to A nucleotide substitution in position 938 of *kfoC *which corresponds to an amino acidic missense mutation from arginine to glutamine at position 313 of K4CP (Fig. 3). We named this mutation R-313-Q.

To check whether R-313-Q was responsible for the chondroitin increase in strain VZ15, we transferred a plasmid bearing either the VZ15 *kfoC *gene or the wt *kfoC *in a "clean" K4 wt background and the two merodiploid strains were analyzed for their chondroitin production. Yield (g/gcdw) of the strain carrying extra-copies of the wt *kfoC *was 0.162, while that of the isogenic strain carrying the VZ15 *kfoC *was 0.271. This experiment allowed us to conclude that mutation R- 313-Q is directly involved in the increase in chondroitin production in strain VZ15, regardless from hypothetical additional mutations due to the MNNG treatment.

### Docking analysis of K4CP and its ligands

K4CP is a bi-functional enzyme with two different active sites: site A1, at the N-terminal domain, adds GalNAc (*N*-acetyl-D-galactosamine) units to chondroitin chains, whereas site A2, at the C-terminal domain, adds GlcUA (D-glucuronic acid) units [20]. Osawa and co-workers obtained crystal structures of K4CP in which the A site was binding either UDP-GalNAc in a catalytically productive orientation or UDP-GlcUA in a non-productive orientation [20]. This finding has suggested that UDP-GlcUA may act as a regulator/inhibitor of the A1 active site [20].

Residue Arg-313 is located near the A1 site with the guanidine moiety at about 7-8 Å from the acetyl moiety of GalNAc and the carboxyl moiety of GlcUA. Hence, substitution of the positively charged arginine residue with a neutral residue, like glutamine, could impair selectively the binding of the negatively charged GlcUA to active site A1, thus reducing the hypothesized inhibitory effect of UDP-GlcUA.

In order to verify this hypothesis we docked UDP-GalNAc and UDP-GlcUA to the A1 site of wild type and mutant K4CPs. The docking analysis showed that Arg-313 provides a significant direct electrostatic contribution of -2.5 kcal/mol^-1 ^to the UDP-GlcUA/K4CP binding energy, that is lost in mutants with neutral residues at position 313 (Table 1).

**Table 1:** Energy parameters for the model complexes UDP-GlcUA/K4CP, UDP-GlcUA/(R313Q)-K4CP, UDP-GalNAc/K4CP and UDP-GalNAc/(R313Q)-K4CP.

Complex	Conf. of Arg268^a^	Energy (kcal/mol)						
		
		LPE^b^	LE^b^	Δ**E^c^**	R268^d^	X313^d^	D311^d^	S334^d^
**K4CP/UDP-GlcUA^e^**	**ext.^a^**	**-102.8**	**-64.1**	-5.4	**-12.7**	**-2.5**	**2.2**	**-0.8**
	bent^a^	-99.3	-64.0		-8.7	-2.5	2.0	-2.0

**(R313Q)/UDP-GlcUA^e^**	ext.	-100.6	-63.8	+1.3	-12.6	0.14	2.0	-1.4
	**bent**	**-96.5**	**-63.9**		**-8.0**	**0.21**	**1.8**	**-1.9**

**K4CP/UDP-GalNAc (R313Q)/UDP-GalNAc**	bent	-100.6	-71.5	n.a.^f^	-4.3	-0.64	0.55	-0.72
	bent	-99.4	-71.5	n.a.^f^	-3.7	~0	0.59	-0.72

^a ^Conformation of Arg-268: extended (ext.), χ^1 ^= 61°, χ^2 ^= -165°, χ^3 ^= 176°, χ^4 ^= -163°; bent, χ^1 ^= 55°, χ^2 ^= 175°, χ^3 ^= 168°, χ^4 ^= -86°.

^b ^LPE and LE are, respectively, the ligand/protein interaction energy and the ligand internal energy (including torsional energy and van der Waals and electrostatic interactions among ligand atoms).

^c ^Difference between the total energy of the complex with Arg-268 in the extended conformation and the total energy of the complex with Arg-268 in the bent conformation.

^d ^Contributions of single residues to LPE.

^e ^The energy parameters of the most stable form of each complex are shown in bold.

^f ^n.a., not applicable.

**Figure 4:**
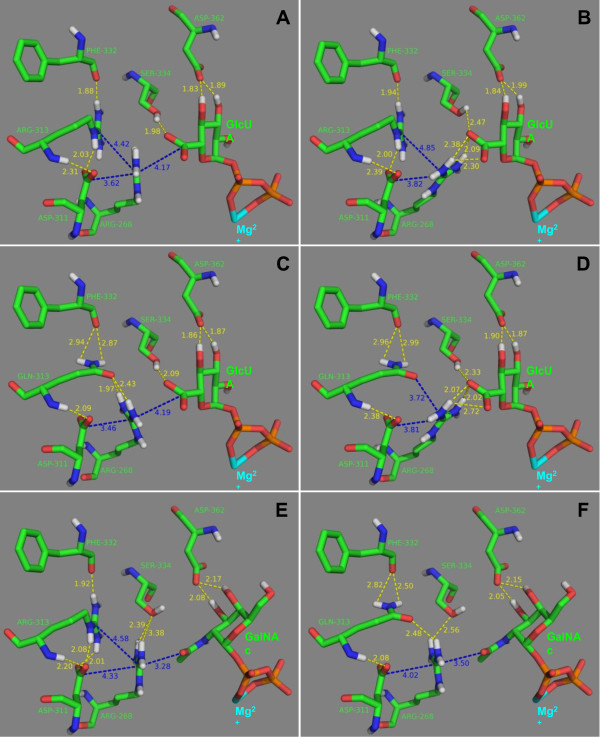
**Docking analysis**. Model complexes UDP-GlcUA/K4CP with Arg-268 in the bent (A) and the extended (B) conformation, UDP-GlcUA/(R313Q)-K4CP with Arg-268 in the bent (C) and the extended (D) conformation, UDP-GalNAc/K4CP (E), and UDP-GalNAc/(R313Q)-K4CP (F). Carbon atoms are colored green, nitrogen atoms blue, oxygen atoms red, phosphorus atoms orange, magnesium ion cyan and hydrogen atoms white. For clarity, only polar hydrogen atoms are shown. H-bonds are shown as yellow dashed lines, whereas other distances are shown as blue dashed lines. Distances are in Å.

An indirect contribution may also derive from the conformation of the adjacent residue Arg-268 (Fig. 4). This residue can adopt a partially bent conformation with the guanidine moiety close to Asp-311 and Arg-313 (Fig. 4A) and an extended conformation with the guanidine moiety at H-bond distance from the carboxyl moiety of GlcUA (Fig. 4B). In the extended conformation the binding energy of UDP-GlcUA is considerably higher due to the stronger interaction between Arg-268 and the carboxylate group of glucuronic acid (Fig. 4B). This strong interaction and the lower electrostatic repulsion between Arg-313 and Arg-268 contribute to make the complex UDP-GlcUA/K4CP with Arg-268 in the extended conformation about 5 kcal/mol more stable than the same complex with Arg-268 in the bent conformation.

When Arg-313 is substituted by glutamine, the guanidine moiety of Arg-268 forms strong H-bonds with the carbonyl oxygen of Glu-313 side-chain (Fig. 4C). Therefore, in this case, the complex with Arg-268 in the bent conformation (Fig. 4C) is slightly more stable than the complex with Arg-268 in the extended conformation (Fig. 4D). As a consequence the R-313-Q mutation decreases the UDP-GlcUA/K4CP interaction energy (LPE) by about 6 kcal/mol (Table 1).

On the contrary, mutation R-313-Q does not impair the binding of the substrate, GalNAc, as the direct contribution of Arg-313 to the UDP-GalNAc/K4CP binding energy is low (about 0.6 kcal/mol). Moreover, the binding of GalNAc to the A1 active site requires Arg-268 in the bent conformation (Fig. 4E-F) which is stabilized by Gln-313. Therefore, the R-313-Q mutation decreases the UDP-GalNAc/K4CP binding energy by only 1.2 kcal/mol (Table 1).

## Conclusions

Our results indicate that a mutation (R-313-Q) in the structural gene *kfoC *for chondroitin polymerase (K4CP) of *E. coli *K4 present in mutant VZ15 is responsible for a significant increase of chondroitin production by this strain. The docking analysis revealed that the A1 site of K4CP, binds UDP-GalNAc with an affinity comparable to that of the wild type K4CP, whereas the UDP-GlcUA interaction with the mutant A1 site is strongly reduced. Our data, in addition to providing original information about the mechanism of K4CP enzyme activity, also points to mutant VZ15 as a promising tool towards a cheap and efficient fermentative production of chondroitin for pharmaceutical preparations.

## Methods

### Materials, medium and microorganisms

Glycerol, ampicillin, IPTG (Isopropyl β-D-1-thiogalactopyranoside) and all the nutrients used to prepare the culture medium were from Sigma (St. Louis, MO, USA) except the soya peptone that was from OXOID (Basingstoke, Hampshire, UK). All the reagents and the glucuronic acid standard used for the carbazole assay as well as the chemicals used for preparation of running buffer and washing solutions for HPCE (High performance capillary electrophoresis) analysis were analytical grade and from Sigma (St. Louis, MO, USA).

The strain used in all experiments was *E. coli *K4 serotype O5:K4:H4 (U141, 11307), a class II microorganism that is sensitive to ampicillin and kanamycin. It was purchased from the Culture Collection University of Goteborg (Goteborg, Sweden). Wild type K4 and mutants were stored in 20% glycerol at -80°C.

The growth medium reported by Rodriguez (16) for *E. coli *K4 was modified by changing the carbon and nitrogen sources in the following manner (K4 medium):10 g·L^-1 ^of glycerol, 1 g·L^-1 ^of soya peptone, 2 g·L^-1 ^of KH_2_PO_4_, 9.7 g·L^-1 ^of K_2_HPO_4_, 0.5 g·L^-1 ^of Na_3_C_6_H_5_O_7_, 1 g·L^-1 ^of (NH_4_)_2_SO_4 _and 0.1 g·L^-1 ^of MgCl_2_.

### Random mutagenesis by MNNG

MNNG (*N*-methyl-*N*'-nitro-*N*-nitrosoguanidine) was used at 70 μg/ml, and the procedure was essentially as follows. Two samples of 2 ml each from an *E. coli *K4 culture grown up to 0.4-0.5 O.D. were centrifuged at 6,000 × g for 3'. The bacterial pellets were re-suspended in 1 ml of K4-medium with and without MNNG and the two suspensions were incubated for 1 h at 37°C. Serial dilutions of treated and untreated (negative control) cells were plated on medium containing ampicillin (2,5 μg/ml) and chloramphenicol (3 μg/ml). Plates were incubated at 30°C to prevent killing of temperature-sensitive mutants.

### indole-3-acetic acid colorimetric assay

Colonies of wild type and MNNG-induced mutants were placed onto nitrocellulose filters (Hybond) and incubated with 0.5% indole-3-acetic acid in ethanol for 10'. Filters were then placed in a box saturated with vapours generated by a 36% HCl solution and incubated for 30'. Colour was developed by reaction of indole-3-acetic acid with fructose residues linked to GlcUA chondroitin monomers.

### Preparation of supernatant samples

K4 polysaccharide production was analysed by carbazole assay and by capillary electrophoresis. In both cases samples were collected after an over night growth and centrifuged at 1.700 × g (J-20 XP, Beckman Coulter, USA) to separate the supernatant from cells. Before being analysed, 2 ml of supernatant were ultra-filtered on 10 KDa centrifugal filter devices (YM-10 Centricon, Millipore, France), dia-filtered with two volumes of bi-distilled water and concentrated again to 1/10 of their initial volume. Samples containing the polysaccharide were then analysed by carbazole assay or capillary electrophoresis.

### Carbazole assay

The glucuronic acid content of the supernatant samples was tested by the carbazole colorimetric assay according to the procedure reported by Dubois et al. [21]. Briefly, 5 ml of a 9.5 g/L sodium tetraborate solution in H_2_SO_4 _were added to 1 ml of the samples in glass tubes and put at 100°C for 15 minutes. After cooling, 0.2 ml of a 1.25 g/L carbazole solution in ethanol were added and tubes were put again at 100°C for 15 minutes. Sample absorbancies were measured at 530 nm. Standard solutions of glucuronic acid in the range from 0.0065 to 0.065 g/L were prepared to built a calibration curve that was used to determine the glucuronic acid content of the samples. The final polysaccharide concentration was obtained multiplying the uronic acid concentration, obtained by the carbazole assay, by a fixed numerical factor (2.78) that takes in consideration the trisaccharide repeated unit composition of K4 capsular chain.

### High performance capillary electrophoresis

Capillary electrophoresis analysis was performed using a HPCE instrument (P/ACE MDQ; Beckman-Coulter, Palo Alto, CA, USA), equipped with a deuterium lamp (from 190 to 600 nm wavelength range) and a photo diode array detector. Runs were performed in electro kinetic mode on an uncoated fused-silica tube (Beckman Coulter, 50 μm I.D., 70 cm of total length, 60 cm of effective length) with an operating buffer containing disodium hydrogen phosphate (40 mM), sodium tetra borate (10 mM) and SDS (40 mM), buffered at pH 9.0 with 1 M HCl, degassed and filtered through a 0.45 μm membrane.

The K4 polysaccharide in the supernatants of shaking flask microbial growths was detected and quantified according to the analytical method, already described [22].

A 60 nL sample total volume, calculated with the Poisseuille equation, was automatically injected, operating in pressure mode for 10 seconds at 1.0 psi, and separations were performed at 25°C, in normal polarity mode applying 20 kV for 30 minutes; the analytes were detected at 190 nm. Sample peak areas were recorded and calculated using the Beckman-Coulter 32 Karat Software while sample concentrations were determined substituting the areas values in the K4 calibration curves. The method showed an high linearity in the range from 0.1 to 3.5 ng·nl^-1 ^for the K4 polysaccharide.

### Polymerase chain reaction (PCR) and cloning

*kfoC *(~2100 bp) amplification was performed by polymerase chain reaction (PCR) using chromosomal DNA of *E. coli K4 *as template and KfoC-Forward (5'-cgatatacagaacaatacg-3') and KfoC-Reverse (5'-gccgggcaacaagggagaccg-3') as primers.

Primers were designed according to the nucleotide sequence of *E. coli *K4, available in gene bank (AB079602).

Reaction mixture was made up of: chromosomal DNA (50 ng); primers F e R (5 μM both); dNTP (0.2 mM); PCR buffer 1× (Invitrogen); MgCl_2 _(1.5 mM); Taq DNA polymerase 5 U (Invitrogen); The PCR programme was as follows: 5' at 96°C, than 30 cycles consisting of 45" at 94°C, 1' at 55°C and 3' at 72°C.

PCR product was cloned into plasmidic vector pGEM-t-Easy to perform the sequence analysis (CRIBI, Padova). pETBlue-1 vector was used to generate the merodiploid K4 derived strains containing extra copies of VZ15 *kfoC *and wt *kfoC *transcribed under the control of *tet *promoter.

### Modelling of substrates into the active site of K4CP

Substrates were docked into the active site of K4CP using the Monte Carlo Energy Minimization strategy. The ZMM-MVM molecular modelling package (ZMM Software Inc.; http://www.zmmsoft.com) [23] was used for all calculations. Atom-atom interactions were calculated using the AMBER force field [24] with a cut-off distance of 8 Å and a relative dielectric constant = 4. Energy calculations included van der Waals, electrostatic, H-bonds, and torsion components. Hydration component was not included. The "Zl" module of ZMM was used to optimize the geometry of substrates and to attribute partial charges. X-ray structures of K4CP bound to UDP-GlcUA and UDP-GalNAc (pdb codes: 2Z86 and 2Z87) were used to build the initial model complexes. To reduce computational time a double-shell model of the enzyme was built. The inner shell included all the residues with at least one side-chain atom at less than 13 Å from UDP-GalNAc or UDP-GlcUA. During energy calculation procedures all the torsion angles of these residues were allowed to vary. The outer shell included all the residues with at least one side-chain atom at less than 22 Å from UDP-GalNAc or UDP-GlcUA. During energy calculation procedures the torsion angles of the outer shell residues were not allowed to vary. The metal cofactor was modelled as a Mg^2+ ^ion with a single water molecule bound at the apical position of a square pyramid as observed in the crystal structures of K4CP. The "atom/atom-distance constraint", available in ZMM, was used to fix the distances between the Mg^2+ ^ion and the surrounding atoms, including the water molecule, the oxygen atoms of the phosphates of the UDP moiety, the atoms of the imidazole ring of His-386 and the carboxylate of Asp-241. A force constant of 1000 kcal/mol/Å was used. Complexes with total energy up to 5 kcal/mol higher than the lowest-energy complex were stored for the analysis of energetic contributions. Total energy was partitioned into intra-receptor, intra-ligand (LE in Table 1), receptor/ligand (LPE in Table 1) and energies of the constraints. Receptor/ligand energy was further partitioned into van der Waals, electrostatic, and H-bonds components. Moreover, receptor/ligand energy was also partitioned (i) per active site residue in order to evaluate the contribution of each residue to the binding of ligands (the last four columns in Table 1), and (ii) per ligand atoms. The pdb files of the initial complexes, and the ZMM instruction files containing the list of mobile residues, constrains and parameters used during calculations are available on request.

The PyMOL software (DeLano Scientific LLC) was used to visualize structures and to prepare figures of the complexes.

## Competing interests

The authors declare that they have no competing interests.

## Authors' contributions

MV and MDF planned the work that led to the manuscript; AZ and OFR produced and analyzed the experimental data; DC participated in the interpretation of the results; EN performed the docking analysis. MV and MDF wrote the paper; CS and MDR participated in the discussion of experimental results and in the revision of manuscript's intellectual content. All authors read and approved the final manuscript.
